# Surface/Interface Chemistry Engineering of Correlated‐Electron Materials: From Conducting Solids, Phase Transitions to External‐Field Response

**DOI:** 10.1002/advs.202002807

**Published:** 2021-01-05

**Authors:** Zejun Li, Qiran Wu, Changzheng Wu

**Affiliations:** ^1^ Hefei National Laboratory for Physical Sciences at the Microscale CAS center for Excellence in Nanoscience and CAS Key Laboratory of Mechanical Behavior and Design of Materials University of Science and Technology of China Hefei Anhui 230026 PR China

**Keywords:** conducting solids, correlated electronic materials, external‐field response, phase transitions, surface/interface engineering

## Abstract

Correlated electronic materials (CEMs) with strong electron−electron interactions are often associated with exotic properties, such as metal‐insulator transition (MIT), charge density wave (CDW), superconductivity, and magnetoresistance (MR), which are fundamental to next generation condensed matter research and electronic devices. When the dimension of CEMs decreases, exposing extremely high specific surface area and enhancing electronic correlation, the surface states are equally important to the bulk phase. Therefore, surface/interface chemical interactions provide an alternative route to regulate the intrinsic properties of low‐dimensional CEMs. Here, recent achievements in surface/interface chemistry engineering of low‐dimensional CEMs are reviewed, using surface modification, molecule−solid interaction, and interface electronic coupling, toward modulation of conducting solids, phase transitions including MIT, CDW, superconductivity, and magnetism transition, as well as external‐field response. Surface/interface chemistry engineering provides a promising strategy for exploring novel properties and functional applications in low‐dimensional CEMs. Finally, the current challenge and outlook of the surface/interface engineering are also pointed out for future research development.

## Graphical abstract

Low‐dimensional correlated electronic materials, with strongly‐coupled interactions between charge, spin, lattice, and orbit, show many exotic properties including metal‐insulator transition (MIT), charge density wave, superconductivity, and magnetoresistance (MR) effects. The latest advances in surface/interface chemistry engineering of low‐dimensional correlated electronic materials, in particular, surface modification, molecule−solid interaction, and interface electronic coupling, toward exotic electronic states and external‐field response are highlighted.

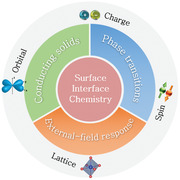

## 1 Introduction

Strongly correlated electron materials (CEMs) exhibit many unusual physical properties, such as metal‐insulator transition (MIT), charge density wave (CDW), superconductivity, and magnetoresistance (MR) effect and so on, appealing for intriguing applications of optoelectronic and spintronic devices.^[^ ^1^, ^2^, ^3^, ^4^ ^]^ These exotic phenomena originate from the coupling interactions of charge, spin, lattice, and orbital degrees of freedom, inducing phase transitions under external fields.^[^ ^5^, ^6^ ^]^ When the dimension of CEMs decreases, such as one‐dimensional (1D) atomic chain structure and two‐dimensional (2D) plane, quantum confinement effect can be brought in these low‐dimensional CEMs nanostructures. Thus, the combination of electronic correlation and quantum confinement effect could bring new physical phenomena and functional applications in low‐dimensional CEMs.^[^ ^2^, ^7^ ^]^ To better understand and use their fascinating properties of electron−electron interactions, many efforts have been devoted to regulating the phase transitions of low‐dimensional CEMs nanostructures.^[^ ^8^, ^9^, ^10^, ^11^ ^]^

Surface/interface chemistry engineering offers an important experimental route for controlling phase transitions in low‐dimensional CEMs nanostructures. It is well known that chemical reactions have congenital advantages including strong chemical bonding, various reaction types and selectivity, and continuous tunability. Also, low‐dimensional CEMs have extremely high specific surface area and expose large amounts of surface coordination‐unsaturated atoms and dangling bonds.^[^ ^12^ ^]^ Therefore, low‐dimensional CEMs can actively react with different electron‐donating and electron‐accepting atoms or molecules via surface coordination and chemical bonding. These chemical interactions would induce charge transfer, spin coupling, and interfacial strain, which can effectively adjust the electronic and magnetic properties of low‐dimensional CEMs.^[^ ^13^ ^]^ Furthermore, these surface/interface engineering can preserve their pristine lattice and intrinsic many‐body behaviors, without introducing substitutional disorder, which is expected to trigger new physical phenomena and create novel phases of matter. Besides, due to electronic correlations, local surface/interface modulation can alter the whole electronic states of low‐dimensional CEMs. More importantly, the entangled order parameters of charge, spin, lattice, and orbital in low‐dimensional CEMs would bring about collaborative modulation, which means that controlling one order parameter has an associated influence on other parameters. In a word, with the advantages of chemical interactions and electron‐correlation characteristics, surface/interface engineering could be a powerful strategy to controllably regulate electronic states in low‐dimensional CEMs, which is favorable to trigger novel physical properties in low‐dimensional correlated systems.

**Figure 1:**
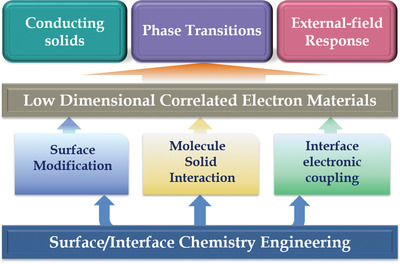
a) Schematic illustration of surface/interface chemistry engineering, including surface modification, molecule−solid interaction and interface electronic coupling, for modulating the conductivity, phase transitions, and external‐field response of low‐dimensional correlated electronic materials (CEMs).

In this progress report, we focus on a series of the latest advances on surface/interface chemistry engineering to control the electronic phases of low‐dimensional CEMs, including electronic conductivity, phase transitions, and their response driven by external fields (as illustrated in **Figure** **1**). We primarily introduce the surface modification, molecule−solid interaction and interface electronic coupling, which successfully realize regulation of the conducting solids, MIT, CDW, superconductivity, and magnetism transition, as well as the infrared modulation and magneto‐electronic response. These results demonstrate the possibility of manipulating macroscopic phase and external‐field response applications by surface/interface modulation. Finally, based on the current achievements of the surface/interface engineering, some discussions and outlook for future research are presented.

## 2 Surface/Interface Chemistry Engineering of Conducting Solids

**Figure 2:**
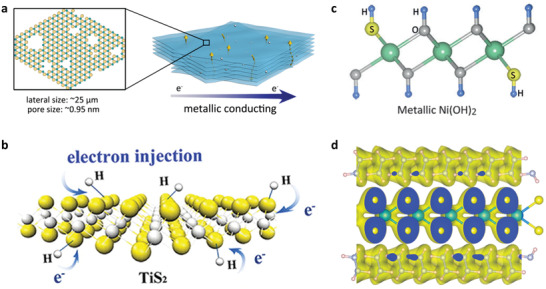
a) Acid‐assisted exfoliation of LiTaS_2_ single crystal achieved conductive TaS_2_ monolayers with large lateral size. Reproduced with permission.^[^ ^14^ ^]^ Copyright 2018, American Chemical Society. b) Schematic illustration of surface hydrogen atoms incorporation on TiS_2_ nanosheets. Reproduced with permission.^[^ ^16^ ^]^ Copyright 2013, American Chemical Society. c) Schematic illustration of the surface sulfur atom modified Ni(OH)_2_ nanosheets by reacted with H_2_S. Reproduced with permission.^[^ ^17^ ^]^ Copyright 2016, Wiley‐VCH. d) Schematic illustration of the charge distribution of TaS_2_−N_2_H_4_ superlattice. Reproduced with permission.^[^ ^19^ ^]^ Copyright 2020, American Chemical Society.

The intrinsic conductivity of low‐dimensional conducting solids is mainly determined by the charge‐carrier density and their transport mobility. For example, large‐sized nanosheets can reduce the contacted boundary, which would facilitate the charge transport and enhance the conductivity of 2D films. Recently, metallic TaS_2_ crystals were successfully intercalated through deliberately controlling lithium content. The lithium intercalated TaS_2_ products were pretreated with acid solution and exfoliated in the deionized water. Due to the gigantic interlayer expansion and etching with acid treatment, metallic TaS_2_ monolayers with tens of microns size and sub‐nanopores were realized.^[^ ^14^ ^]^ The films assembled by these large‐sized TaS_2_ monolayers exhibited much higher conductivity compared with the small‐sized one (**Figure** **2**a). Moreover, doping heterogeneous atoms is generally used to modify the charge carrier density, which is efficient to regulate the energy band structure and electrical behavior of solid materials.^[^ ^15^ ^]^ However, doping atoms with different sizes inevitably tends to alter the local lattice structure and electronic energy level in an uncontrolled manner. The collapsed crystal frameworks suppress their intrinsic properties and make it difficult to build a clear relationship between structure and property of functional materials.

The surface atom incorporation strategy can trigger charge transfer for tuning the intrinsic conductivity of conducting solids, in the meanwhile, maintaining pristine lattice. Especially, hydrogen is the smallest atom, which can donate electrons into the solid structure to regulate carrier density without changing the lattice framework. In this regard, hydrogen incorporated TiS_2_ nanosheets have been achieved by a chemical exfoliation strategy (Figure 2b).^[^ ^16^ ^]^ The modified hydrogen atoms donated electrons into the S−Ti−S lattice to increase the electron density and enhance electronic correlation, leading to an ultrahigh conductivity of 6.76 × 10^4^ S m^−1^ at 298 K for a 2D assembled thin film. Furthermore, surface S modified Ni(OH)_2_ nanosheets were developed by annealing Ni(OH)_2_ nanosheets with H_2_S flow under 130 °C for 1 h (Figure 2c).^[^ ^17^ ^]^ X‐ray absorption fine structure measurements revealed structural model of sulfur substitution of oxygen atoms in the S‐modified Ni(OH)_2_ nanosheets. Compared with the original semiconducting Ni(OH)_2_, the S‐modified Ni(OH)_2_ nanosheets showed metallic behavior, exhibiting excellent conductivity of 3.19 × 10^3^ S m^−1^ at room temperature. Besides, systematic structural characterizations confirmed that the pristine lattice framework of Ni(OH)_2_ nanosheets was preserved after surface S modification.

Besides the surface atom incorporation, surface molecule modification can also be used to regulate the electronic conductivity by controlling charge carrier transport and interfacial charge injection. For example, H_2_O molecule was used to regulate the conductivity of 2D metallic VS_2_ nanosheet films by controlling the planar electron transport.^[^ ^18^ ^]^ In VS_2_ films, electrons transported from the side‐exposed V atoms of an individual nanosheet toward another nanosheet covered below. When the side‐exposed V atoms were absorbed by H_2_O molecules, electrons transport channel was blocked, thus the conductivity substantially decreased with almost two orders of magnitude. Recently, the N_2_H_4_ molecules were used to intercalate the mechanically exfoliated 2H‐TaS_2_ nanosheets from the crystal to form a hybrid superlattice structure.^[^ ^19^ ^]^ Resistance measurement showed that the TaS_2_ nanosheets after intercalation of N_2_H_4_ molecules displayed higher conductivity compared with that of the pristine TaS_2_. To explain this behavior, the charge distribution and differential charge density were calculated, revealing the charge transfter from N_2_H_4_ molecules to the TaS_2_ lattice (Figure 2d). The charge transfer regulated the electronic state and deeply enhanced the density of state (DOS) distribution near the Fermi level of TaS_2_. Therefore, surface/interface chemistry engineering, leading to charge injection and control of charge transport, is a promising approach to tune the conductivity of low‐dimensional solid materials.

## 3 Surface/Interface Chemistry Engineering of Phase Transitions

### 3.1 Metal‐Insulator Transition Modulation

**Figure 3:**
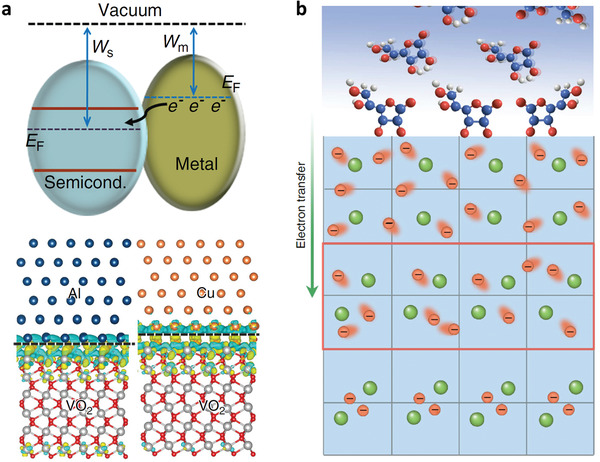
a) Schematic depiction of electrons flowing from metal with lower workfunction to semiconductor with a higher workfunction at the interface.^[^ ^30^ ^]^ Copyright 2018, Nature Publishing Group. b) Schematic of the AA molecules chelated on the surface of VO_2_ nanobeams with electron transfer process.^[^ ^31^ ^]^ Copyright 2017, Nature Publishing Group.

Metal‐insulator transition is a common phenomenon in strongly correlated materials, where the change of electronic structure accompanies substantial resistance variation.^[^ ^20^ ^]^ Vanadium dioxide (VO_2_) is a prototype correlated oxide, which exhibits an abrupt first‐order MIT near room temperature of about 68 °C accompanied by a lattice change from rutile to monoclinic structure, giving potential applications in ultrafast switching techniques, Mottronics and memristors.^[^ ^21^, ^22^, ^23^ ^]^ However, its MIT mechanism is still debated due to the entangled electronic and structural transition, mainly focusing on two viewpoints—Mott correlation and Peierls transition.^[^ ^24^, ^25^, ^26^ ^]^ On the other hand, the MIT temperature (*T*_MIT_) of 68 °C is not favorable to the practical applications. Therefore, in order to understand the MIT mechanism and use its MIT properties, many efforts have been devoted to modulating the MIT of VO_2_ including heterogeneous atom doping, electrolyte gate, pressure, and strain modulation.^[^ ^10^, ^11^, ^12^, ^13^, ^14^, ^15^, ^16^, ^17^, ^18^, ^19^, ^20^, ^21^, ^22^, ^23^, ^24^, ^25^, ^26^, ^27^, ^28^ ^]^ Among these modulation strategies, hydrogenation is an effective way to tune the MIT of VO_2_, which can stabilize the metallic VO_2_ phase to room temperature. However, conventional hydrogenation methods usually require catalysts or high‐temperature condition. For example, hydrogenated VO_2_ nanobeams were realized by a hydrogen spillover method using deposited Au film as the catalyst, which fully hydrogenated VO_2_ exhibited metallic state persisted down to 4.2 K.^[^ ^10^ ^]^ Hydric rutile VO_2_ (R) and hydric VO_2_ (M‐R) have been obtained at room temperature by annealing the hydric paramontroseite VO_2_ precursor at 250 °C and 300 °C, respectively.^[^ ^29^ ^]^ In pursuit of a facile hydrogenation treatment, hydrogenated VO_2_ was realized in an acid solution at ambient condition by placing a low‐workfunction metal (Al, Cu, Ag, Zn, or Fe) on VO_2_ surface.^[^ ^30^ ^]^ The workfunction difference would lead to the electron transfer from metal to VO_2_, which subsequently drives protons in surrounding solution to VO_2_ lattice as a result of electrostatic attraction (**Figure** **3**a). The metal with different workfunction would donate different electron and finally form hydrogenated VO_2_ with different H content. For example, Ag/Cu can induce a lightly H‐doped VO_2_, exhibiting conductive metallic state at room temperature. Whereas, the Al/Zn with lower workfunction can transfer more electrons and form heavily H‐doped VO_2_, which presented insulating behavior. Thus, choosing metal with different workfunction can realize the metal to insulator modulation for the H‐doped VO_2_.

To understand the mechanism of phase transitions, identification of the intermediate states in the phase transition process is a reliable route.^[^ ^24^ ^]^ Unfortunately, these phase transitions usually occur transiently within about femtosecond scale, making it a great challenge to characterize the intermediate states. Thus, it can be expected that stabilization of the intermediate states will be a feasible method to understand the puzzling nature of phase transitions. To this end, a surface molecule coordination route was developed to stabilize a metal‐like monoclinic phase in VO_2_ nanobeam, which is an intermediate state in VO_2_ MIT process.^[^ ^31^ ^]^ As depicted in Figure 3b, l‐ascorbic acid molecules (AA) chelated on the surface of VO_2_ nanobeam, inducing electron transfer from AA molecules to VO_2_ nanobeams. In the metal‐like monoclinic region, the injected electron density was not sufficient to suppress the Peierls distortion, transforming to monoclinic lattice. However, the injected electrons would preferentially accommodate in the *d*_xz_/*d*_yz_ subshells, which could partially suppress the Mott correlation and induce a nonequilibrium metal electronic state. Thus, this stabilized metal‐like monoclinic phase gives an evidence that the MIT of VO_2_ is a cooperative interaction of Mott correlation and Peierls transition.

### 3.2 Charge Density Wave and Superconductivity Modulation

**Figure 4:**
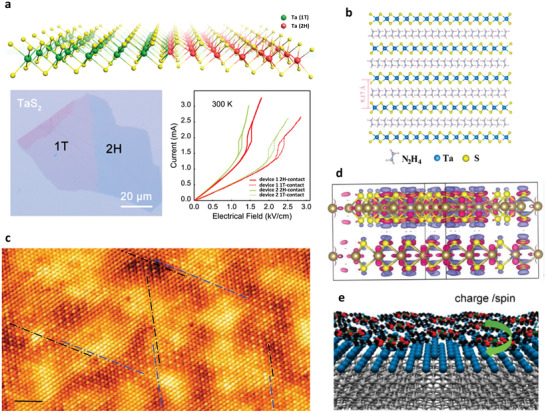
a) Schematic illustration of lateral 1T‐2H TaS_2_ homojunction and the electric field driving charge density wave (CDW) transition with different contacts. Reproduced with permission.^[^ ^36^ ^]^ Copyright 2019, American Chemical Society. b) Schematic structure of the TaS_2_−N_2_H_4_ hybrid superlattice. Reproduced with permission.^[^ ^19^ ^]^ Copyright 2020, American Chemical Society. c) Scanning tunneling microscopy (STM) micrograph (*V*_bias_ = 10 mV, *I*_t_ = 60 pA) of 1T‐Cu_0.07_TiSe_2_ at 1.2 K. Scale bar = 2 nm. Reproduced with permission.^[^ ^40^ ^]^ Copyright 2017, American Physical Society. d) Charge density distribution of twisted TaS_2_. Reproduced with permission.^[^ ^43^ ^]^ Copyright 2017, American Chemical Society. e) Concept of a 2D heterostructure consisting of the superconducting metal atomic layer and self‐assembled organic molecules with charge transfer and local spins to modify the superconducting properties. Reproduced with permission.^[^ ^45^ ^]^ Copyright 2017, American Chemical Society.

Charge density wave is a static modulation of conduction electrons accompanied by a periodic distortion of the material lattice, which usually occurs in linear chain compounds or 2D layered crystals.^[^ ^32^ ^]^ The first‐order CDW phase transition exhibits hysteresis electronic characteristics under external field modulation, providing a great application opportunity in the memristor, neuromorphic circuits, and advanced information storage techniques. For example, 1T‐TaS_2_, a correlated layered compound presenting a successive CDW transition on cooling, has been used to fabricate a memristive switching device controlled by an in‐plane electric field.^[^ ^33^ ^]^ As is known, the electronic contact has a significant effect on the device performance, which gives rise to numerous research about artificial construction of homojunction to reduce the barrier of the electronic contact.^[^ ^34^, ^35^ ^]^ To this end, 1T‐2H‐TaS_2_ homojunction monolayers were successfully fabricated via a solution‐process method (**Figure** **4**a).^[^ ^36^ ^]^ Through precise control of the lithiation procedure for 1T‐TaS_2_ crystals, 1T phase maintained at low Li content, whereas converted to metallic 2H‐TaS_2_ phase at high Li content regions. 1T‐2H‐TaS_2_ homojunction monolayers can be obtained by a programmed exfoliation of this polymorphic crystals with 1T and 2H phases using H_2_O and acids. Then this 1T‐2H‐TaS_2_ homojunction monolayers were fabricated into the device with Au electrode contacted on the 2H phase region. Compared with the device with pure 1T‐TaS_2_ phase, this 1T‐2H‐TaS_2_ homojunction monolayers showed a 50% decrease of the electric field to drive the CDW switching.

On the other hand, CDW is also related to some interesting quantum phenomena, such as superconductivity. The coexistence of CDW and superconductivity has been widely observed in some 2H‐transition metal dichalcogenides (TMDs), such as 2H‐NbSe_2_, 2H‐TaSe_2_, and 2H‐TaS_2_.^[^ ^37^ ^]^ A plentiful of modulation approaches (e.g., press and doping) have demonstrated the reduction of CDW transition temperature would increase the superconducting transition temperature, indicating a competition relationship between them.^[^ ^38^ ^]^ Recently, Guo et al. reported N_2_H_4_ molecules intercalated 2H‐TaS_2_ hybrid superlattice, which the electronic transport measurements demonstrated an enhanced superconductivity and suppression of the CDW transition (Figure 4b).^[^ ^19^ ^]^ The theoretical calculations showed the charge transfer from N_2_H_4_ molecules to 2H‐TaS_2_ lattice increased the density of states (DOS) near the Fermi level, hence suppressing the CDW and enhancing the superconductivity.

Moreover, superconductivity can also be induced in TMDs with CDW by controlling the charge‐carrier density through doping or gating.^[^ ^37^, ^39^ ^]^ For example, pristine 1T‐TiSe_2_ exhibits a commensurate 2 × 2 × 2 CDW at T_CDW_ of about 200 K. The transport measurements showed that the Cu intercalated TiSe_2_ (Cu_x_TiSe_2_) gradually suppressed the CDW and superconductivity appeared when the Cu content was more than 0.04, which also suggested a competition between CDW and superconductivity.^[^ ^38^ ^]^ However, an inconsistent viewpoint about the influence of Cu intercalation on the CDW in Cu_x_TiSe_2_ was provided by a scanning tunneling microscopy (STM) study.^[^ ^40^ ^]^ Figure 4c showed the STM image for the Cu_0.07_TiSe_2_ sample at 1.2 K. According to the transport measurements, at 1.2 K, this Cu_0.07_TiSe_2_ sample was in the superconducting state while the CDW phase was no longer detected. However, from the STM image, it can be seen that the CDW was not completely suppressed, but existed as some short‐range ordered CDW domains. This result suggested that the suppression of CDW may not be the necessary prerequisite for the appearance of superconductivity in Cu_x_TiSe_2_. Therefore, this STM result supported another opinion that superconductivity may result from the inhomogeneities of the CDW pattern.^[^ ^39^, ^41^ ^]^

Regulating the superconducting temperature (*T*_c_) is favorable to study the underlying mechanism and practical applications. To increase the *T*_c_, pioneering methods mainly relied on the doping or applying pressure. Subsequently, high‐*T*_c_ superconductivity was observed at the interface of metallic and insulating copper oxides,^[^ ^42^ ^]^ triggering the research interest in the interface effects on superconductivity. Pan et al. found an enhanced superconductivity in the restacked 2H‐TaS_2_ nanosheets, in which the *T*_c_ increased to 3 K compared with the 0.8 K of pristine TaS_2_.^[^ ^43^ ^]^ The restacked TaS_2_ nanosheets were obtained from the layer‐by‐layer assembling of the free‐standing TaS_2_ nanosheets via vacuum filtration, which produced twisting structure with the same interlayer distance as that of the pristine TaS_2_. The transport measurement showed that the restacked TaS_2_ nanosheets presented higher superconducting *T*_c_ (3 K) and the CDW transition was not observed in the measured temperature range. The density functional theory (DFT) calculations demonstrated that the twisted TaS_2_ structure exhibited reduced symmetry and more delocalization of DOS near the Fermi surface (Figure 4d), thus enhancing the superconductivity.

Besides the above inorganic interface electronic modulation, the assembled organic molecules with charge and spin effects are also effective candidates to modulate the superconducting *T*_c_. The flexibility of organic molecules on the surface of superconductors allows for a fine modulation of the local interactions at molecule‐superconductor interface.^[^ ^44^ ^]^ Yoshizawa et al. presented a controlled modulation of superconductivity by choosing different organic molecule assembling layers.^[^ ^45^ ^]^ They modified two kinds of metal phthalocyanines (MnPc and CuPc) on the superconducting In atomic layer. Transport measurements showed that these two metal phthalocyanines exhibited opposite effects on the *T*_c_, which the MnPc decreased the *T*_c_ and CuPc enhanced the *T*_c_. They deemed this distinctive behavior was due to the different electronic and magnetic states for these two molecules (Figure 4e). The increased *T*_c_ for CuPc can be understood by the hole doping to the In layer with a negligible magnetic moment interaction. For MnPc, although the hole doping occurred, its strong magnetic interaction with the In conduction electron would suppress the superconductivity due to its d_z_^2^, d_xz_, and d_yz_ orbitals extending out of the plane.

### 3.3 Magnetism Transition

**Figure 5:**
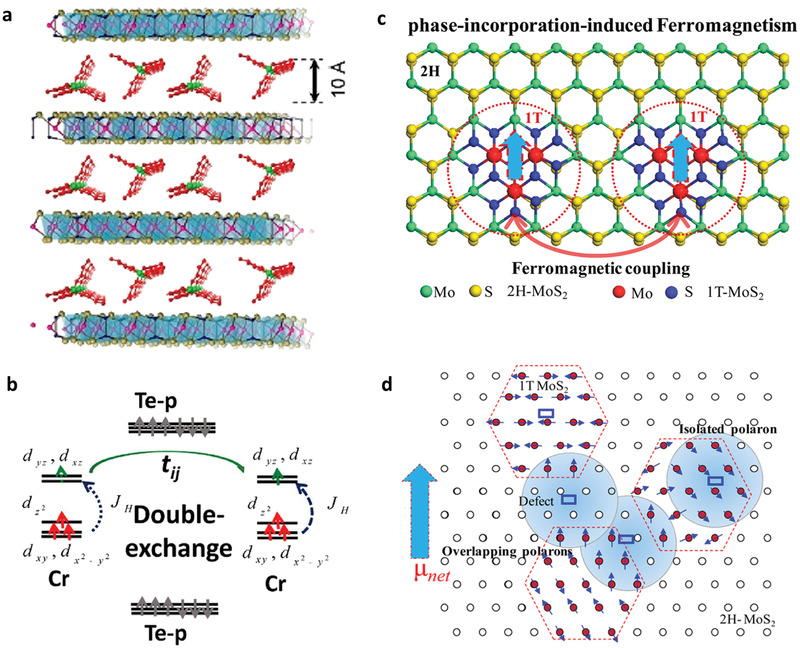
a) Schematic structure model of (TBA)Cr_2_Ge_2_Te_6_. b) Schematic diagrams of the double‐exchange interaction in (TBA)Cr_2_Ge_2_Te_6_. a,b) Reproduced with permission.^[^ ^50^ ^]^ Copyright 2019, American Chemical Society. c) Diagrammatic representation of the phase incorporation strategy to achieve ferromagnetism of 2H‐MoS_2_ nanosheets. d) Representation of magnetic polarons for explaining the ferromagnenism in 1T@2H‐MoS_2_ nanosheets. c,d) Reproduced with permission.^[^ ^52^ ^]^ Copyright 2015, American Chemical Society.

Recent emerging 2D van der Waals ferromagnetic materials have attracted many attentions, which have great potential in future advanced spintronic devices.^[^ ^46^ ^]^ In the magnetic van der Waals crystals, the magnetic order and Curie temperature behave a strong layer‐dependent characteristics. For example, in the van der Waals magnetic semiconductor CrI_3_, its monolayer and trilayer maintained ferromagnetism while the bilayer CrI_3_ showed an antiferromagnetism.^[^ ^47^ ^]^ By using the scanning Kerr microscope, Gong et al. found that the Curie transition temperature of Cr_2_Ge_2_Te_6_ displayed a monotonic increase with increasing thickness from ≈30 K in the bilayer to about 68 K in the bulk.^[^ ^48^ ^]^. Also, their magnetic properties including saturation magnetization, Curie temperature, and coercive force can be manipulated by tuning the carrier density through a field effect transistor devices.^[^ ^49^ ^]^ Recently, a facile and feasible approach had been developed to regulate the magnetism and electronic state of the van der Waals ferromagnetic Cr_2_Ge_2_Te_6_ semiconductor.^[^ ^50^ ^]^ Through an electrochemical intercalation method, the tetrabutyl ammonium (TBA^+^) cations were intercalated into the interlayers of Cr_2_Ge_2_Te_6_ to form the (TBA)Cr_2_Ge_2_Te_6_ (**Figure** **5**a). The electronic and magnetization measurements showed that the (TBA)Cr_2_Ge_2_Te_6_ exhibited metallic behavior and obviously increased Curie temperature from 67 K to 208 K compared with the pristine Cr_2_Ge_2_Te_6_ semiconductor. The theoretical calculations revealed that the electrochemical intercalation of TBA^+^ cations could induce electron doping to Cr_2_Ge_2_Te_6_ layers, thus transforming from semiconductor to metal. The enhanced Curie temperature was attributed to the change from weak superexchange interaction in pristine Cr_2_Ge_2_Te_6_ to a strong double‐exchange interaction in (TBA)Cr_2_Ge_2_Te_6_ (Figure 5b). These results demonstrated the intercalation of organic ions would be an efficient method to tune the electronic state and magnetism of van der Waals magnets.

The surface modulation can also transform an intrinsic nonmagnetic materials to become ferromagnetism. For example, 2H‐MoS_2_ is a nonmagnetic material, hindering its application in the spintronic devices. Although theoretical calculations suggested that strain and vacancy doping can induce ferromagnetism in 2H‐MoS_2_ nanosheets,^[^ ^51^ ^]^ the question was the induced ferromagnetism was not stable and tended to recover to the nonmagnetic state under external treatments. To realize a stable ferromagnetism in MoS_2_ nanosheets, Cai et al. developed a phase incorporation strategy, which successfully induced a robust room‐temperature ferromagnetism in MoS_2_ nanosheets (Figure 5c).^[^ ^52^ ^]^ The phase incorporated MoS_2_ nanosheets were synthesized by a two‐step reaction method. The initial 2H‐MoS_2_ nanosheets were obtained by a hydrothermal reaction from (NH_4_)_6_Mo_7_O_24_·4H_2_O and thiourea. Then the as‐obtained 2H‐MoS_2_ nanosheets were dispersed in ethanol solution and autoclavated again at 220 °C to promote the formation of S vacancy (V_S_). These V_S_ can transform the surrounding lattice of 2H‐MoS_2_ into 1T phase, thus giving rise to the creation of phase incorporated MoS_2_ (1T@2H‐MoS_2_) nanosheets. The observed robust ferromagnetism in the 1T@2H‐MoS_2_ nanosheets can be understood from the bound magnetic polaron (BMP) model. According to the HRTEM image, the highest distance distribution between two neighboring 1T‐MoS_2_ regions is 1–3 nm, which is smaller than the polaron (<3 nm). The spins of the localized V_S_ could align the magnetic moments of the nearby Mo ions in 1T‐MoS_2_ phase, producing effective magnetic field to activate the ferromagnetic exchange interactions between Mo ions within the magnetic polaron (Figure 5d). It can be expected that such a surface vacancy engineering provides an effective tool to tune the electronic phase and magnetism in nanostructure materials.

## 4 Surface/Interface Chemistry Engineering of Interaction Between Superconductivity and Magnetism

### 4.1 Yu−Shiba−Rusinov Bound State

The interplay and competition of superconductivity and magnetism have always been the hot research topic in condensed matter physics. Abrikosov and Gor'kov ever predicted that a magnetic perturbation would reduce the superconducting order parameter and induce a quasiparticle excitation within the superconducting gap.^[^ ^53^ ^]^ For example, an individual magnetic atom adsorbed on the surface of s‐wave superconductors can induce discrete spin‐polarized states inside the superconducting energy gap, which is called the Yu−Shiba−Rusinov (YSR) bound state. In 1997, Ali Yazdani et al. directly probed the local electronic properties of a superconductor in the vicinity of a single magnetic atom using STM, evidencing the presence of localized YSR quaiparticle excitations within the superconducting gap.^[^ ^54^ ^]^ Later, the YSR bound states were also observed in STM studies of single magnetic adatoms and molecules deposited on 3D superconducting Pb and Nb crystals.^[^ ^55^, ^56^ ^]^

**Figure 6:**
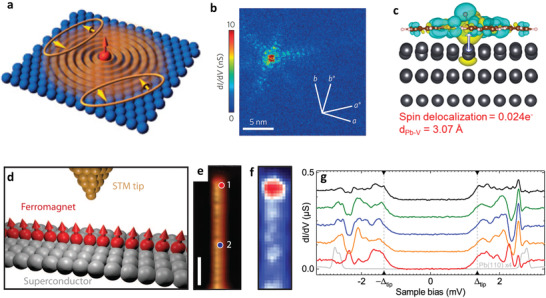
a) Calculated behaviour of a Yu−Shiba−Rusinov (YSR) bound state in an isotropic s‐wave superconductor with 2D electronic band structure. b) Conductance map taken at *V* = −0.05 meV, showing a few star‐shaped structures created by localized magnetic impurities at zero magnetic field. a,b) Reproduced with permission.^[^ ^57^ ^]^ Copyright 2015, Nature Publishing Group. c) Density functional theory (DFT) calculations for oxygen‐up vanadyl phthalocyanine (VOPc) on Pb, compressed VOPc with additional tilt of vanadium‐oxygen (VO) by 30° toward the Pc plane. Reproduced with permission.^[^ ^59^ ^]^ Copyright 2018, American Chemical Society. d) Schematic of the proposal system for Majorana quasi‐particle (MQP) bound states: a ferromagnetic atomic chain is placed on the surface of strongly spin−orbit coupled superconductor and studied using scanning tunneling microscopy (STM). e) Scanning tunneling microscopy (STM) spectra measured at the end and middle of the atomic chain. f) Spatial and energy‐resolved conductance maps of Fe atomic chain at zero bias. d−f) Reproduced with permission.^[^ ^61^ ^]^ Copyright 2014, American Association for the Advancement of Science. g) dI/dV spectra acquired with a superconducting tip on the bare surface and on a long Co chain.^[^ ^62^ ^]^ Copyright 2017, American Chemical Society.

Nevertheless, it should be noted that these observed YSR states extended only a few atomic scales, which is not favorable to the remote coupling of magnetic systems through the superconducting state. To enhance the spatial extent of the YSR bound states, Ménard et al. analyzed the YSR states in 2D superconducting system based on Rusinov's theory, and found that the YSR states induced in 2D superconductor can spatially extend orders of magnitude longer than that in the 3D case (**Figure** **6**a). To evidence their prediction, they probed the YSR states of individual Fe impurities on layered 2H‐NbSe_2_ superconductor, which was known for exhibiting 2D electronic character.^[^ ^57^ ^]^ As expected, the YSR bound states around individual Fe impurities were characterized by a six‐pointed star‐shaped pattern as far as tens of nanometers on the spectroscopic map (Figure 6b), which showed greatly enhanced spatial extension compared with previously observed YSR bound states in 3D superconductors. Thus, the YSR state in 2D superconductors behaved a large distance from the impurity. They deemed that such a long‐range YSR pattern was attributed to the 2D character of 2H‐NbSe_2_ and strong electronic coupling for the embedded Fe impurities in the NbSe_2_ lattice.

The YSR bound states are induced by the exchange scattering at magnetic adsorbates on the surface of superconductors. Its binding energy is dependent on the exchange coupling *J* between the adatom and superconducting substrate.^[^ ^58^ ^]^ It can be expected that, depending on the strength of exchange coupling, a weak interaction would result in a free‐spin ground state and a strong interaction would localize quasiparticle state at the magnetic impurities. The earlier experiments can only obtain discrete exchange coupling strength between the adatoms and the superconductors due to the fixed adsorption site for magnetic atoms. To realize a continuous controlling of the exchange coupling between the magnetic impurities and superconductors, using a magnetic molecule as the magnetic adsorbate was proposed.^[^ ^59^ ^]^ The flexibility of the magnetic molecules showed tunable exchange coupling interaction, where the noncovalent interface with the superconductor can be mediated through the ligand shell. As such, the vanadyl phthalocyanine (VOPc) molecules were deposited on the Pb superconducting surface, which exhibited two adsorption configurations: oxygen pointing toward the vacuum (oxygen‐up molecules) and oxygen pointing toward the Pb (oxygen‐down molecules). Initially, the oxygen‐up molecules showed no YSR bound states due to the weak molecule spin‐superconductor interaction. By moving the STM tip toward this oxygen‐up molecules and applying mechanical force onto the VO center, the YSR bound state can be smoothly induced. Thus, the reduction of tip‐molecule distance can enhance the coupling between molecular spin and the superconductor. It also found that the binding energy of YSR state gradually reduced with the approach of STM tip and a sharp inversion of the peak asymmetry near zero bias energy, indicating a quantum phase transition. DFT calculations suggested that mechanism force from the STM tip gave rise to a gradual tilt of the VO bond toward the phthalocyanine ligand. The tilt can effectively move the magnetic orbital out of the Pc plane, making more effective spin delocalization toward the Pb surface and realizing a continuously tunable spin‐superconductor interaction (Figure 6c). Thus, the surface molecule modification provides a tunable coupling interaction to induce exotic properties in correlated materials.

### 4.2 Majorana Fermions

Majorana fermion is an elementary particle with the intriguing property of being their own antiparticle proposed by Ettore Majorana, which can be used in the future topological quantum computer. In 2013, two theorists predicted that Majorana bound state can appear at the edges of a hybrid system consisting of a chain of magnetic atoms coupled to a superconductor with strong spin−orbital interaction.^[^ ^60^ ^]^ They proposed that the strongly entangled magnetic moments and electrons can induce a magnetic spiral structure, which can drive the system into a topological superconducting phase supporting Majorana fermions. Subsequently, Nadj−Perge et al. fabricated a ferromagnetic Fe atomic chains on the surface of a superconductor Pb (Figure 6d).^[^ ^61^ ^]^ Spin‐polarized STM studies showed the evidence of ferromagnetism on the Fe chains and strong spin−orbit coupling with the superconducting Pb surface. High‐resolution spectroscopy showed a sharp zero‐bias peak (ZBP) at about 10–20 Å away from the ends of the chain. The spectroscopic maps clearly presented the localized nature and spatially modulated decay of the ZBPs at one end of the Fe chain (Figure 6e,f), which was a principal experimental evidence for the predicted Majorana bound state in a topological superconductor. In addition, by using a superconducting tip, the spectroscopic maps exhibited the same ZBP features for Majorana bound state at the end of the chains as that with the normal STM tip, and the signature of Majorana bound state was greatly suppressed in the middle of the chains. In a word, these observations gave an important evidence of the edge‐bound Majorana fermions in the magnetic atomic chains on a superconductor.

Due to this possible realization of Majorana zero modes in the Fe chains on superconducting Pb surface, Ruby et al. further studied the Co chains on the same Pb surface to check whether topological superconductivity and Majorana modes can be observed.^[^ ^62^ ^]^ Co atomic chains were deposited by e‐beam evaporation from a cobalt rod onto the clean surface of Pb crystal. A Co‐coated STM tip was used to detect the magnetism of the Co chains, a ferromagnetic state can be determined by a uniform difference contrast of the dI/dV map along the Co chain for spin‐up and spin‐down tip. The dI/dV spectrum on the Co chain showed broad resonances within the superconducting gap, indicating the YSR bound states arising from the exchange coupling between spin‐polarized Co *d* states and the Pb substrate. The hybridization of the YSR states of neighboring Co atoms along the chain resulted in the spin‐polarized bands. This ferromagnetic chain with spin‐polarized bands on a *s*‐wave superconductor provided a possible system to realize the topological superconductivity and Majorana zero modes. A superconducting Pb tip was used to explore the Majorana zero modes. Although a resonance was observed at zero energy (Figure 6g), however, this zero‐energy signal was presented along the whole chain but not localized at the end of the chain. The Majorana bound states were expected to be localized on an atomic‐scale distance. The absence of localization for the zero‐energy resonance in the Co chains indicated that the observed features cannot be originated from the Majorana mode. The theoretical calculations showed that this hybrid Co chain system had an even number of Fermi points within half the Brillouin zone, which cannot meet the requirement to form a topological phase and thus suppress the Majorana zero modes.

### 4.3 Coexistence of Superconductivity and Ferromagnetism

Conventional superconductivity is a quantum state of matter where the Cooper pairs condense from the electrons with opposite spin direction and moment.^[^ ^63^ ^]^ Ever since the discovery of superconductivity, the interplay between superconductivity and magnetism is the central topic in solid‐state materials, appealing for future quantum computing and superconducting spintronics.^[^ ^64^, ^65^, ^66^ ^]^ Unfortunately, the exchange field of ferromagnetic order aligns the electron spins and raises the kinetic energy of electrons via Zeeman and orbital effects, intending to break the Cooper pairs and suppress superconductivity.^[^ ^67^ ^]^ Thus, it has been generally considered that the ferromagnetism cannot coexist with superconductivity. In fact, the coexistence of superconductivity and ferromagnetism in natural crystals is rare. Much attention has therefore been devoted to artificially coexisting systems. Such coexistence can be typically prepared by integrating composite structures with intrinsically superconducting and ferromagnetic materials. For example, constructing ferromagnetism/superconductor multilayer structure, self‐assembly periodically interleaved with superconducting [TaS_2_]^−0.33^ anions and ferromagnetic [M^II^_0.66_M^III^_0.33_(OH)_2_]^+0.33^ cations using their electrostatic interactions.^[^ ^68^ ^]^ However, there remains a question that whether the coexistence of superconductivity and ferromagnetism can be designed in a single freestanding nanomaterials, expecting for stronger interplay of superconducting and ferromagnetic orders.

**Figure 7:**
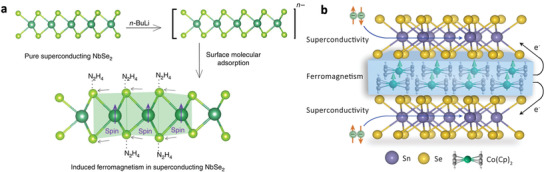
a) Schematic illustration of the coexistence of superconductivity and ferromagnetism in 2D NbSe_2_ nanosheets by surface molecular adsorption. Reproduced with permission.^[^ ^69^ ^]^ Copyright 2016, Nature Publishing Group. b) Superconductivity and ferromagnetism in 2D SnSe_2_−Co(Cp)_2_ superlattice by molecule‐confined engineering. Reproduced with permission.^[^ ^70^ ^]^ Copyright 2017, American Chemical Society.

To deal with this challenge, a surface‐molecule modulation strategy was put forward for the incorporation of short‐range ferromagnetic domains into single freestanding superconducting NbSe_2_ nanosheets.^[^ ^69^ ^]^ As is known, pristine NbSe_2_ is a superconductor but nonmagnetic, because the covalent Nb–Se interaction stifles magnetic moment of Nb^4+^ ions. In this work (**Figure** **7**a), through the electrostatic interactions between negatively charged NbSe_2_ nanosheets and the adsorbed polar reductive hydrazine (N_2_H_4_) molecules, a local structure distortion was realized while the pristine structure was reserved. This local structure distortion led to the elongated covalent Nb–Se bonds and attenuation of their covalent interactions, thus successfully yielding short‐range ferromagnetism in superconducting NbSe_2_ nanosheets. The ferromagnetic regions and superconducting regions in the hydrazine‐treated NbSe_2_ nanosheets were spatially separated, which the ferromagnetism originated from the distorted NbSe_2_ lattice and the superconductivity was from the normal NbSe_2_. Compared with pure NbSe_2_ sample, hydrazine‐treated NbSe_2_ nanosheets showed slight decrease of superconducting transition temperature and the behavior of susceptibility in the normal state was temperature‐dependent. This work provides a molecule modulation of 2D structure for achieving the integration of ordered magnetism and superconductivity in a single 2D materials.

In view of the prominence of these efforts, a fundamental question arises if such coexistence can be imprinted into a freestanding structure with nonsuperconducting and nonferromagnetic components. Enhanced interplay of superconductivity and ferromagnetism can be expected due to the coupled interactions between these two nonsuperconducting and nonferromagnetic components. Recently, a molecule‐confined engineering was demonstrated in 2D SnSe_2_−Co(Cp)_2_ organic−inorganic superlattice, bringing freestanding coexistence of superconductivity and ferromagnetism originated from interlayered interactions between nonsuperconducting and nonferromagnetic building blocks.^[^ ^70^ ^]^ It is known that the layered SnSe_2_ host material is a nonmagnetic semiconductor, and pristine Co(Cp)_2_ is a paramagnetic molecular crystal. Strikingly, as Co(Cp)_2_ molecules were intercalated into SnSe_2_ interlayers, flatly lying Co(Cp)_2_ molecules in strongly confined SnSe_2_ interlayers weakened the coordination field, triggering spin transition to high‐spin state with ferromagnetic behavior (Figure 7b); meanwhile, cyclopentadienyls of Co(Cp)_2_ molecules strongly coupled with Se−Sn−Se lattice, leading to electron transfer into SnSe_2_ layers to generate superconductivity. This unusual coexistence of superconductivity and ferromagnetism led to a remarkable coupled interaction between the molecular ferromagnetic layers and inorganic superconducting layers. The molecule bounded in confined space by chemical tailoring provides an efficient path to induce novel correlated electronic properties in 2D hybrid materials.

## 5 Surface/Interface Chemistry Engineering of External‐Field Response Materials

Low‐dimensional CEMs are very sensitive to external disturbances, such as heat, light, electric field, and magnetic field, giving rise to significant potential in application of external‐field response devices. When the related energies are close to each other, any small perturbation can effectively change the equilibrium relationship between the competing phases and induce phase transitions in low‐dimensional CEMs. These phase transitions are directly associated with the distinctive change of electrical, magnetic, and optical properties. Thus, low‐dimensional CEMs can be superior candidates as external‐field response materials that sense external stimuli by monitoring the change of their intrinsic physical properties.

### 5.1 Infrared Light Response

In strongly correlated electron systems, MIT is an intriguing physical phenomenon often accompanied by dramatic changes in optics and magnetism, which has great potential for use in advanced response devices.^[^ ^71^ ^]^ However, at present, the MIT of most materials is not near room temperature, which is unsuitable for practical applications.

**Figure 8:**
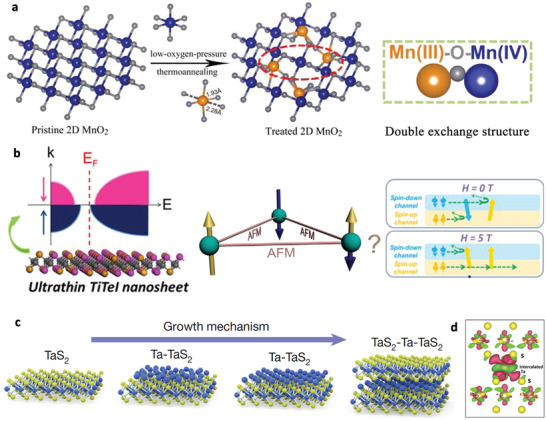
a) MnO_2_ nanosheets with Mn(III)−O−Mn(IV) double‐exchange structure. Reproduced with permission.^[^ ^72^ ^]^ Copyright 2017, American Chemical Society. b) Magnetoresistance (MR) effect induced by anionic solid solution strategy in spin‐frustrated TiTeI nanosheets. Reproduced with permission.^[^ ^80^ ^]^ Copyright 2014, American Physical Society. c) Schematic depicting the layer‐by‐layer growth of Ta‐intercalated TaS_2_ bilayer. d) Contour plot of charge density difference in Ta‐intercalated Ta_7_S_12_ bilayer. c,d) Reproduced with permission.^[^ ^89^ ^]^ Copyright 2020, Nature Publishing Group.

Recently, double exchange effect was introduced in 2D MnO_2_ nanosheets, which successfully realized the MIT of MnO_2_ nanosheets near room temperature accompanied with infrared modulation.^[^ ^72^ ^]^ As illustrated in **Figure** **8**a, pristine MnO_2_ nanosheets were heated at 100 °C for 30 min and further up to 220 °C for 2 h in N_2_ atmosphere. The low‐oxygen pressure annealing induced Mn^3+^ ions with lower valence state in the MnO_2_ nanosheets and produced double‐exchange structure of Mn^3+^‐O^2−^‐Mn^4+^ with neighboring Mn^4+^ and O^2−^ ions. The treated MnO_2_ nanosheets showed an obvious MIT near room temperature arising from the double exchange effects. In order to study the infrared modulation of MnO_2_ nanosheets, in situ infrared spectroscopy (FTIR) has been used to detect the sensitive response in different magnetic fields at room temperature. It was worth noting that the infrared transmittance of the treated MnO_2_ nanosheets increased with rising measured temperature under the constant magnetic field. In addition, the infrared transmittance was reversible during heating and cooling at 1000–2000 cm^−1^, making it a promising platform to construct advanced infrared smart devices.

Besides the modulation of infrared transmittance, infrared light harvesting has also attracted much attention for promising applications in infrared imaging^[^ ^73^ ^]^ and infrared detection^[^ ^74^ ^]^. In order to better keep tracking photons, photoactive materials with a narrow bandgap are essential for converting infrared photons into electric signals with high quantum efficiency.^[^ ^75^ ^]^ As a correlated material, VO_2_ (M) nanobeam possesses unique advantages to be the optimal candidate for infrared detection, including the narrow bandgap (0.58 eV) and high surface‐to‐volume ratio which will lead to significant sensitivity to environmental perturbation. Nonetheless, pure VO_2_ (M) nanobeam is disadvantaged by easy recombination of the photogenerated excitons.^[^ ^76^, ^77^ ^]^ According to theoretical calculation, VO_2_ (M)‐V_2_O_5_ heterointerface can form type II heterojunction, which would efficiently promote the separation of excitons to improve infrared detection performance. In this regard, VO_2_ (M)/V_2_O_5_ core–shell nanobeam heterostructures were obtained by controlled oxidation of monoclinic VO_2_ (M) nanobeams in air at 400 °C with different annealing time. Surface V^4+^ was oxidized to V^5+^ to form V_2_O_5_ and the inner still kept VO_2_ (M) phase.^[^ ^78^ ^]^ Benefited from the well‐defined type II heterointerface, ultrahigh responsivity of 2873.7 A W^−1^ and specific detectivity of 9.23 × 10^12^ Jones at the 990 nm infrared light were achieved at room temperature, which were comparable to traditional materials including heavy metals. This finding paves a new way to design oxide heterostructures for intriguing applications in optoelectronic nanodevices.

### 5.2 Magnetoelectronic Response

MR devices, in which electrical resistance can be controlled in a material in response to an external magnetic field, has revolutionized the sensitivity of magnetic read heads, through manipulation of the spin and charge degrees of freedom.^[^ ^79^ ^]^ With the confined electronic structures and various lattice configurations, low‐dimensional correlated nanomaterials have great potential for realizing giant MR effects.

An anionic solid solution process was proposed that introduce intrinsically net spin in 2D TiTe_2_ nanosheets, regulating the electronic property and leading to a negative MR effect (Figure 8b).^[^ ^80^ ^]^ Because of the perfect size compatibility, I^−^ can easily replace the anionic Te^2−^ of TiTe_2_ to form a TiTe_2−x_I_x_ anionic solid solution, inducing electronic phase transition from metallic TiTe_2_ to semiconducting TiTeI. The anion substitution induced considerable Ti^3+^ formation with unpaired 3d^1^ electron, leading to frustrated antiferromagnetic Ti^3+^ magnetic moments, which resulted in spin‐dependent scattering of the conducting electrons on the local magnetic moments. The value of MR for the ultrathin TiTeI nanosheet reached as high as −85% (5 T, 10 K). Recently, solid solutions of TMD have attracted wide attention, unique electronic and optical properties have been reported.^[^ ^81^, ^82^, ^83^, ^84^, ^85^ ^]^ Furthermore, a half‐metallic structure of TiSe_2_ nanosheets was successfully developed by a dual‐native‐defects engineering of Ti‐atom incorporation and Se‐anion defects.^[^ ^86^ ^]^ This half‐metallic TiSe_2_ nanosheets resulted in a high‐spin‐polarized current and local magnetic moments, exhibiting a large negative MR with a value of −40% (5T, 10 K). When electron transport is confined along 1D channel, strong magnetoelectronic response can be expected. In this regard, a hydric effect was exploited to regulate the spin configuration of V−V atomic chains in VO_2_ nanowires, resulting in a large negative MR in VO_2_ (M) nanowires.^[^ ^87^ ^]^ The hydrogen‐treated VO_2_ (M) nanowires introduced V^3+^ (3d^2^) ions into the zigzag V−V chains, triggering ferromagnetic‐coupled V^3+^‐V^4+^ dimers to produce 1D superparamagnetic VO_2_ nanowires. Due to the spin‐polarized electron hopping between these ferromagnetic V^3+^‐V^4+^ dimers, ‐33.3% negative MR value was achieved at room temperature under 500 Oe.

Besides the above routes, intercalation of the foreign atoms into the van der Waals (vdW) gap is a common strategy to regulate the magnetic and electronic properties of layered correlated materials. For example, pristine 2H‐TaS_2_ is a nonmagnetic layered TMD material with a superconductivity at about 0.8 K. When the Fe atoms were intercalated into its interlayer to form Fe_0.28_TaS_2_ crystals, ferromagnetism was induced and the superconductivity was suppressed.^[^ ^88^ ^]^ In addition, such a Fe_0.28_TaS_2_ crystals exhibited a large MR exceeding 60% at 2 K under an out of plane magnetic field. The question is these traditional intercalation methods are difficult to form a long‐range crystalline unless using very harsh treatment. In contrast with the foreign atom intercalation, the self‐intercalation using the native atoms into TMDs may produce local energy minima in the intercalation phase diagram. To this end, recently, Zhao et al. reported a self‐intercalation of native atoms into bilayer TMDs, which showed tunable magnetism in the Ta‐intercalated TaS_2_ bilayers.^[^ ^89^ ^]^ The Ta‐intercalated TaS_2_ bilayers were obtained by molecular beam epitaxy (MBE) deposition through controlling the initial Ta‐to‐S flux ratio (Figure 8c). A Ta:S flux ratio of about 1:6 produced a√3a × √3a superlattice of Ta atoms with the chemical stoichiometry of Ta_7_S_12_, while a Ta:S ratio around 1:8 and 1:5 resulted in the Ta_9_S_16_ and Ta_10_S_16_, respectively. The magneto‐transport measurements revealed a ferromagnetic order in the Ta_7_S_12_ sample, which a linear MR up to 9 T was observed at low temperature. Also, a nonlinear Hall effect was observed ascribed to the anomalous Hall effect, arising from ferromagnetism in conductors. DFT calculations revealed the ferromagnetism of self‐intercalated Ta_7_S_12_ can be induced by the double‐exchange mechanism due to the charge transfer from intercalated Ta atom to the TaS_2_ layer (Figure 8d). The intercalated Ta atoms introduced additional spin‐split bands across the Fermi level to form a magnetic ground state, which the magnetic moments were localized on the *d* orbitals of the intercalated Ta atom. This self‐intercalated modification provides a new approach to tune the electronic and magnetic properties of layered vdW materials.

## 6 Conclusions and Outlook

In summary, we have reviewed the latest advances in surface/interface chemistry engineering of low‐dimensional CEMs nanostructures toward conducting solids, phase transitions, and external‐field response in recent years. We have specifically introduced the surface modification, molecule−solid interaction, and interface electronic coupling methods, successfully achieving control of conductivity and various intriguing phase transitions. On the basis of sensitive response of low‐dimensional CEMs to external fields, prototype devices were constructed to investigate their electronic and optical response with magnetic field and light. Although rapid progress has been made on the study of surface/interface chemistry engineering of low‐dimensional CEMs nanostructures, there are still some issues that deserve future study. For example, in many cases, the link between the host and guest is not clear for these surface and interface modification, more direct characterization can be used to determine the interacting force, bonding mode, and molecular geometry and so on. It is also important to study and develop rational surface/interface engineering strategies to create novel physical properties and functional applications of low‐dimensional CEMs nanostructures.

## Conflict of Interest

The authors declare no conflict of interest.
